# The influence of membrane physical properties on microvesicle release in human erythrocytes

**DOI:** 10.1186/1757-5036-2-7

**Published:** 2009-08-24

**Authors:** Laurie J Gonzalez, Elizabeth Gibbons, Rachel W Bailey, Jeremy Fairbourn, Thaothanh Nguyen, Samantha K Smith, Katrina B Best, Jennifer Nelson, Allan M Judd, John D Bell

**Affiliations:** 1 Department of Physiology and Developmental Biology, Brigham Young University Provo, Utah 84602, USA

## Abstract

Exposure of human erythrocytes to elevated intracellular calcium causes fragments of the cell membrane to be shed as microvesicles. This study tested the hypothesis that microvesicle release depends on microscopic membrane physical properties such as lipid order, fluidity, and composition. Membrane properties were manipulated by varying the experimental temperature, membrane cholesterol content, and the activity of the trans-membrane phospholipid transporter, scramblase. Microvesicle release was enhanced by increasing the experimental temperature. Reduction in membrane cholesterol content by treatment with methyl-β-cyclodextrin also facilitated vesicle shedding. Inhibition of scramblase with R5421 impaired vesicle release. These data were interpreted in the context of membrane characteristics assessed previously by fluorescence spectroscopy with environment-sensitive probes such as laurdan, diphenylhexatriene, and merocyanine 540. The observations supported the following conclusions: 1) calcium-induced microvesicle shedding in erythrocytes relates more to membrane properties detected by diphenylhexatriene than by the other probes; 2) loss of trans-membrane phospholipid asymmetry is required for microvesicle release.

PACS Codes: 87.16.dj, 87.16.dt

## Introduction

We have recently reported the use of fluorescence spectroscopy to generate a pseudo phase map of physical properties of erythrocyte membranes as a function of temperature and membrane cholesterol content [1]. These experiments used three fluorescent probes that differ slightly in the properties they detect based on companion experiments with artificial membranes of defined composition. The result was the equivalent of a phase diagram mapping properties such as lipid order, spacing, and fluidity for intact human erythrocytes. These studies were complemented by a preliminary effort using the enzyme phospholipase A_2 _to determine whether there was any functional significance to the various regions of membrane behaviors observed by the probes [2]. The success of that initial attempt has prompted us to pursue additional erythrocyte membrane processes to assess their relationship to membrane properties. We report here our investigations of the relationship between microvesicle release and the pseudo phase map.

Microvesicle shedding is stimulated in human erythrocytes by sustained elevation of intracellular calcium [3]. Other events associated with calcium influx in erythrocytes include: opening of calcium-dependent potassium channels, leading to potassium ion efflux [4,5]; activation of calcium-dependent proteases, including calpain [3]; activation of the phospholipid transporter scramblase, leading to exposure of phosphatidylserine in the outer leaflet of the bilayer [6,7]; cytoskeletal alterations [8-12], a change in erythrocyte morphology from the normal discoid shape to a spherical shape; and an increase in order of membrane lipids as detected by the fluorescent probe laurdan [3,13].

Apparently, several of these calcium-dependent events are involved in the mechanism of microvesicle production and shedding. For example, convincing evidence argues that activation of both calpain and the calcium-dependent potassium channel is critical for vesicle release [3,4,14,15]. Cytoskeletal and membrane proteins such as synexin, a protein involved in bilayer fusion, are also likely to be involved [16]. Studies in platelets have suggested that tyrosine dephosphorylation may regulate proteins involved in the process [17,18]. Although several lines of evidence suggest that translocation of phosphatidylserine is also required, other data have implied the opposite conclusion [15,19-21]. To our knowledge, a potential role for membrane biophysics in microvesicle release has not yet been explored. Nevertheless, the distinct characteristics of microvesicle membranes, such as enrichment with lipid rafts, support this hypothesis [16,22].

To investigate the contributions of membrane composition and biophysical properties to calcium-regulated microvesicle release, we altered membrane cholesterol content, temperature, and the activity of scramblase in human erythrocytes treated with ionomycin, a calcium ionophore. We compared the results quantitatively to prior measurements of membrane properties in pseudo phase maps constructed as a function of temperature and cholesterol content [1]. Phosphatidylserine exposure was manipulated using the pharmacological agent R5421, a specific inhibitor of scramblase [23].

## Materials and methods

### Materials

The intracellular calcium indicator indo-1 (acetoxymethyl ester form), laurdan, and the Amplex^® ^Red cholesterol assay kit were purchased from Invitrogen (Carlsbad, CA). Ionomycin was obtained from Calbiochem (La Jolla, CA) and methyl-β-cyclodextrin (MBCD) and quinine were purchased from Sigma-Aldrich (St. Louis, MO). R5421 was a generous gift from Jeffrey T. Billheimer at Dupont Merck Research Laboratory (Wilmington, DE). All other reagents were obtained from standard sources. Ionomycin, R5421, laurdan, and indo-1 were dissolved in dimethylsulfoxide (DMSO). Quinine was dissolved in ethanol. Stocks of all other reagents were prepared as aqueous solutions.

Erythrocytes were obtained from blood samples obtained during physical exams at the Brigham Young University Student Health Center. The samples were fresh or stored up to 2 days at 4°C in EDTA vacutainers from which patient identification was removed. Control experiments comparing fresh blood with stored samples demonstrated that these storage conditions did not influence the results [3].

Erythrocytes were isolated and washed by centrifugation as described previously [3], resuspended to the original hematocrit (hct) in a balanced salt buffer (NaCl = 134 mM, KCl = 6.2 mM, CaCl_2 _= 1.6 mM, MgCl_2 _= 1.2 mM, Hepes = 18.0 mM, glucose = 13.6 mM, pH 7.4, 37°C). To verify that results were due to transport of calcium (and not magnesium) by ionomycin, experiments were repeated in a variant of this medium in which the typical calcium and magnesium salts were replaced with different concentrations of CaCl_2_. The minimum amount of CaCl_2 _sufficient to produce a full response was 0.6 mM. Under that condition, the results were indistinguishable regardless of which medium was used; hence, the data obtained with both media were pooled. The ionomycin concentration for all experiments was 300 nM.

### Extraction of membrane cholesterol

Washed erythrocytes were suspended in buffer at 0.15% hct and incubated in the presence or absence of varying concentrations of MBCD (0, 0.1, 0.3, 0.6, or 1.0 mM) for 30 min at 37°C. Cells were washed and re-suspended in fresh medium for experiments. The amount of cholesterol extracted from the cells was quantified using the Amplex^® ^Red cholesterol assay kit (Invitrogen, Carlsbad, CA) on methanol/chloroform extracts of samples as described [1].

### Fluorescence spectroscopy and light scattering

Steady-state fluorescence (indo-1) and sample light scatter intensity and noise were monitored using photon-counting spectrofluorometers (Fluoromax-1 and Fluoromax-3 from Jobin Yvon, Edison, NJ, and PC1 from ISS, Champaign, IL). All three instruments maintain sample homogeneity by continuous gentle stirring with a magnetic stir bar. Control experiments (not shown) showed that stir bar speed did not influence light scatter noise. Sample temperature was controlled and maintained using circulating water baths and equilibrated for at least 5 min to achieve temperature stability. Simultaneous assessment of fluorescence intensity at multiple excitation and emission wavelengths was obtained by rapid sluing of monochromator mirrors using control software provided with the instrument. Band pass was set at 4 nm for both monochromators in each case.

Intracellular calcium concentrations at 25°C, 37°C and 45°C were assessed using indo-1 as explained previously [24]. Erythrocyte samples were prepared as described above and further diluted to 1% hct. The acetoxymethyl ester of indo-1 (final concentration = 2.5 μM) was added, and the samples were incubated 60 min at 37°C in a shaking water bath. Cells were washed, resuspended in fresh buffer, placed in cuvette (0.075% hct) and incubated at the indicated temperature in the fluorometer sample chamber. Fluorescence was continuously monitored (excitation = 350 nm, emission = 405 and 480 nm). A baseline was established for 3 min, followed by the addition of ionomycin, and then continued data acquisition for several min.

Phosphatidylserine exposure on the outer leaflet of the cell membrane was assayed by measuring the fluorescence intensity of dansylarginine-N-(3-ethyl-1,5-pentanediyl)amide (3 μM final, excitation = 335 nm, emission = 545 nm) as described [3,25]. This method detects phosphatidylserine by monitoring the rate at which it converts prothrombin to thrombin. A positive control was obtained by the addition of thrombin (2.7 μM).

For light scatter experiments, cell suspensions (0.075% hct) were incubated at the indicated temperature as for fluorescence. Monochromator settings were 500 nm for excitation and 510 nm for emission. As with indo-1 fluorescence measurements, light scatter data were gathered before and continued after ionomycin addition.

## Results

**Figure 1:**
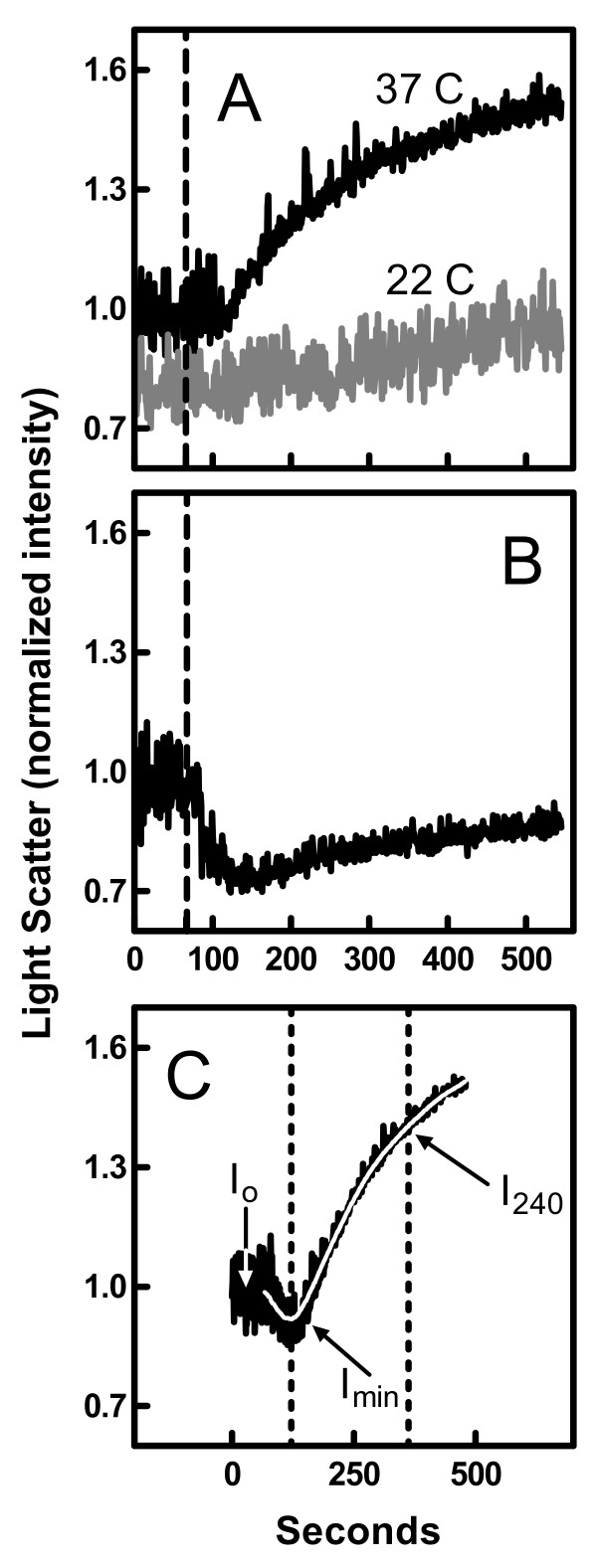
**Effect of ionomycin on light scattered by erythrocytes suspensions**. Panel A: Erythrocytes were treated with ionomycin (added at the dashed line) at 22°C or 37°C. The data are normalized to the average intensity prior to addition of ionomycin. Panel B: The 37°C experiment of Panel A was repeated in the presence of 1 mM quinine. Panel C: The experiment of Panel A was repeated at 43°C. Ionomycin was added at time zero. The dotted lines indicate the minimum in intensity (*I*_*min*_) and the time 240 s later at which *I*_*240 *_is evaluated. The other parameters relevant to Eq. 2 are indicated in the figure. The white curve was obtained by nonlinear regression of the data using Eq. 1.

As shown in Figures 1A and 1C, the addition of ionomycin to human erythrocytes in the presence of calcium caused an increase in the intensity of light scattered by the sample concurrent with a decrease in the signal noise at 37 and 43°C. At 22°C, these effects were reduced or absent. Previous studies have demonstrated that the elevated scattering intensity results from an increase in sample turbidity due to microvesicle shedding [3]. Accordingly, when an inhibitor of microvesicle release was included in the experiment (quinine, see Ref. [5]), the rise in light scatter intensity was greatly reduced (Figure 1B). Instead, the intensity of scattered light decreased because of the calcium-stimulated transition in erythrocyte shape from discocyte to the smaller rounded form termed "spherocyte" [3]. In some cases, the release of microvesicles appeared to be slower than the shape transition leading to an initial drop in light scatter intensity followed by a latent rise (Figure 1C). Previous studies have demonstrated that the reduction in scatter noise also reflects the transition of erythrocyte shape [3,26].

The light scatter data were quantified as illustrated in Figure 1C. First, the time profile was fit by nonlinear regression to an arbitrary function (white curve):

(1)

where *I(t) *is the light scatter intensity as a function of time, *I*_0 _is the initial intensity prior to addition of ionomycin, and *A, B, C, D*, and *E *are arbitrary coefficients. The amount of microvesicle release (*M*) was estimated by calculating the maximum displacement in scatter intensity.

(2)

This displacement was standardized to a time frame of 240 s (since vesicle release appears continuous) and normalized to *I*_*0 *_to account for variations in sample hematocrit and instrument sensitivity. *I*_*min *_and *I*_*240 *_are defined as shown in Figure 1C; *I*_*min *_is the minimum intensity calculated from the regression to Eq. 1 and *I*_*240 *_is the intensity 240 s after the time corresponding to *I*_*min*_.

**Figure 2:**
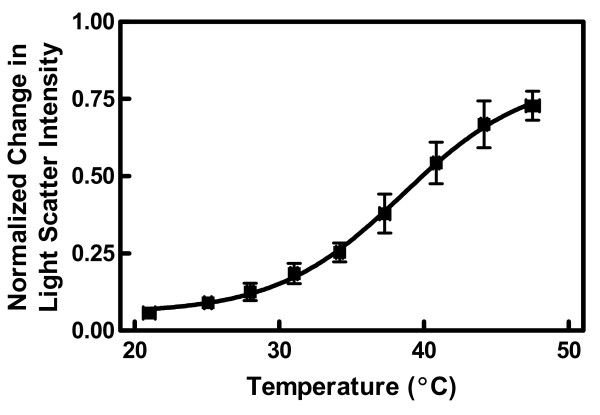
**Effect of temperature on light scatter intensity**. The experiments shown in Figure 1A were repeated at multiple temperatures between 20 and 50°C. Experiments were analyzed for light scatter intensity displacement (*M*) (an index of microvesicle release) using Eqs. 1 and 2. Data points were grouped from several similar temperatures and expressed as mean ± SEM in both dimensions (n = 8–15). The data were fit by nonlinear regression using an arbitrary function. The 95% confidence interval of the inflection point was 35.0–42.0.

The effect of temperature on microvesicle release (Eq. 2) is summarized in Figure 2. The change in intensity (*M*) was minimal and equal to a value of approximately zero at the lowest temperatures. High temperature sensitivity was observed in the range of 30 to 45°C. The inflection point for the effect of temperature occurred at 38.5 ± 3.5°C (midpoint ± 95% margin of error).

**Figure 3:**
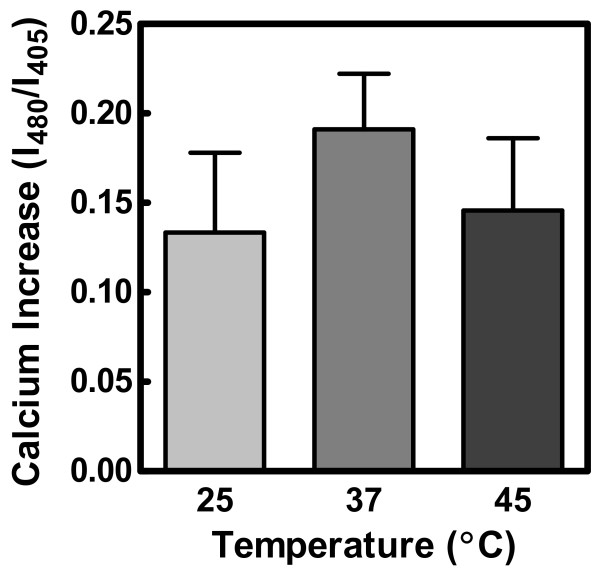
**Effect of temperature on calcium transport by ionomycin**. Data were obtained using indo-1 to monitor internal calcium concentration (see Materials and Methods). Calcium concentration was estimated by calculating the ratio of fluorescence emission intensities at 480 to 405 nm. Data were analyzed by one-way analysis of variance (*p *= 0.71, *n *= 2–3) and are expressed as mean ± SEM.

In order to establish that the observed temperature dependence was not due to differences in the ability of ionomycin to transport ions from the extracellular environment, we used the fluorescent probe indo-1 to report relative internal calcium ion concentration at 25°C, 37°C, and 45°C. There was no statistical difference in the level of calcium achieved after 240 s (the time range covered by the experiments of this study, Figure 3). This result was further validated by examining longer time courses (600–3000 s, data not shown).

**Figure 4:**
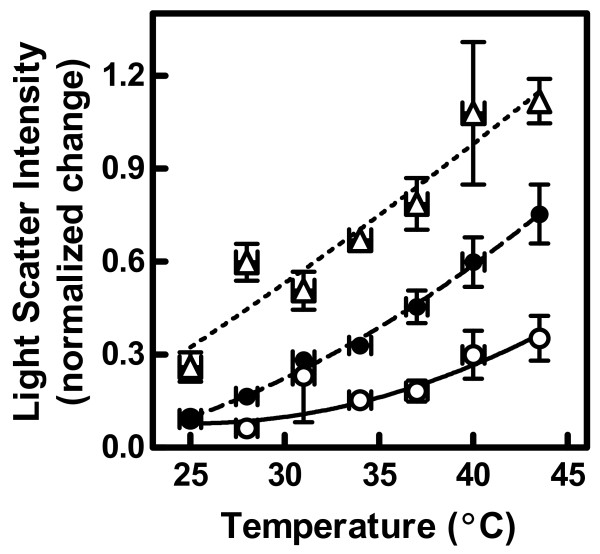
**Effect of membrane MBCD treatment on the temperature dependence of light scatter intensity**. The experiments of Figure 2 were repeated in samples from which membrane cholesterol had been depleted by MBCD (see Materials and Methods). Based on previous calculations [1], samples contained 46.8% (untreated, open circles), 40.7% (treated with 0.3 mM MBCD, solid circles), or 19.0% (treated with 1.0 mM MBCD, open triangles) membrane cholesterol. Data were analyzed as in Figure 2 and expressed as mean ± SEM (n = 3–4). The effects of MBCD concentration and temperature were both significant by two-way ANOVA (*p *< 0.0001 in both cases, *p *= 0.04 for interaction). Two additional concentrations of MBCD not shown in the figure (0.1 mM or 44.9% cholesterol and 0.6 mM or 33.0% cholesterol) were included in the analysis of variance.

To assess the effect of cholesterol on calcium-stimulated microvesicle release, we repeated the experiments of Figure 1 over a range of temperatures from 24 to 45°C (as in Figure 2) before and after depletion of membrane cholesterol (Figure 4). Removal of cholesterol by MBCD treatment resulted in greater differences in light scatter intensity before and after the addition of ionomycin. Two-way analysis of variance showed that both MBCD treatment and temperature had a significant effect on the results (*p *< 0.0001 for both), and that the interaction between the two was also significant (*p *= 0.02, n = 3–4). This interaction between temperature and MBCD concentration confirmed statistically the visual appearance of the data, suggesting that manifestation of the effect of cholesterol depletion occurs to a greater extent at the higher temperatures.

**Figure 5:**
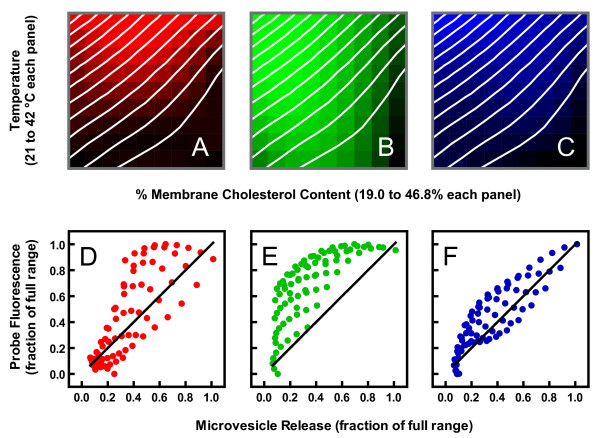
**Contour plot of microvesicle release superimposed on phase maps of membrane properties observed previously with the fluorescent probes merocyanine 540, laurdan, and diphenylhexatriene**. The white contour lines represent increments of 0.08 normalized units of light scatter intensity change (same units as Figure 4). Contour lines were obtained by a two-step nonlinear regression as described previously [1]. Data analogous to those displayed in Figure 4 were first fit to arbitrary functions (*e.g*. polynomials) with curves for each cholesterol concentration and with temperature as the independent variable. The idealized values obtained from these regressions were then fit again with cholesterol concentration as the independent variable and separate curves for each temperature. The optimized values from the second regression were then used to generate a 3-dimensional surface with the Z-axis (intensity) oriented perpendicular to the plane of the figure. These contour plots were superimposed on phase maps for erythrocytes reported previously [1]. Panel A: Merocyanine 540 fluorescence intensity; increasing brightness indicates increasing intensity (from 0.64 to 0.89 normalized units). Panel B: Laurdan fluorescence generalized polarization (GP, see Ref. [1] for details); increasing brightness indicates decreasing GP (from 0.26 to 0.022 GP units). Panel C: Diphenylhexatriene anisotropy; increasing brightness indicates decreasing anisotropy (from 0.24 to 0.18 anisotropy units). Panels D-F: The values used to generate the phase maps ("probe fluorescence") were plotted against the values used to generate the white contour lines ("microvesicle release") for merocyanine 540 (D), laurdan (E), and diphenylhexatriene (F). The solid lines represent perfect correlation for reference (not a linear regression).

By varying both parameters, we were able to calculate a two-dimensional profile of ionophore-induced vesicle shedding and illustrate it as a contour plot in Figure 5 (white lines). The contour plot is shown overlaying pseudo phase maps of membrane properties generated in an earlier investigation in which cholesterol and temperature were similarly varied [1]. The different panels in the figure represent properties assessed with merocyanine 540 (Panel A), laurdan (Panel B), and diphenylhexatriene (Panel C). Although these three probes detect interrelated physical behaviors, studies with artificial membranes of defined composition have made it clear that observations with them are not redundant, and that they are differentially sensitive to various parameters. For example, merocyanine 540 intensity appears most sensitive to interlipid spacing in the headgroup region along the plane of the membrane [1,27-29]. In Figure 5A, increased color brightness denotes greater spacing (increased emission intensity). Laurdan emission spectral shape detects changes in the amount of water invading the bilayer, which tracks closely with lipid order [1,29-32]. In Figure 5B, increased brightness reflects increased disorder (shift of emission spectrum toward longer wavelengths). Diphenylhexatriene anisotropy is also very sensitive to lipid order since movement of the probe in the membrane is mostly determined by physical constraints imposed by the phospholipid acyl chains. Nevertheless, it appears to detect additional lipid movement that laurdan spectra do not sense [1,29,33,34]. For the purposes of this paper, we refer to this additional sensitivity as "fluidity" since it distinguishes solid-ordered and liquid-ordered phases in artificial membranes [1,29]. In Figure 5C increased brightness corresponds to increased fluidity and disorder (reduced anisotropy). Visual inspection of the overlaid data suggests that the pattern of microvesicle release as a function of both temperature and membrane cholesterol content best matches the pattern of diphenylhexatriene fluorescence.

The visual observations in Figures 5A–C were confirmed by correlation analysis (Figures 5D–F). Diphenylhexatriene anisotropy was the fluorescence parameter showing the highest correlation with and least systematic deviation from the level of microvesicle release (Figure 5F; r^2 ^= 0.63 for merocyanine 540, 0.56 for laurdan, and 0.75 for diphenylhexatriene). Multiple regression yielded similar results. When all three pseudo phase maps were considered simultaneously, only laurdan and diphenylhexatriene fluorescence were significant predictors of the rate of microvesicle shedding (*p *< 0.008 in each case). Nevertheless, diphenylhexatriene explained a greater proportion of the variation in vesicle release rates than laurdan (41% *versus *19%). These results suggest that membrane fluidity is the property upon which the ability of ionomycin to induce microvesicle release depends most.

**Figure 6:**
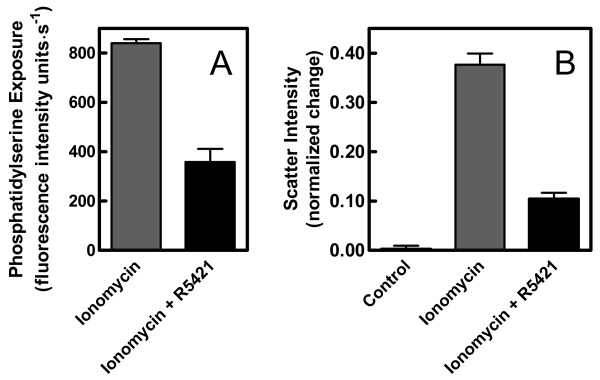
**Effect of the scramblase inhibitor R5421 on ionomycin-stimulated microvesicle release**. Panel A: Phosphatidylserine migration to the outer leaflet of the cell membrane was assessed by fluorescence spectroscopy as described in Materials and Methods. Cells were treated with ionomycin in the presence or absence of 50 μM R5421 (using DMSO as the control solvent for R5421). Panel B: The 37°C experiment of Figure 1A was repeated with erythrocyte samples incubated for 10 min without or with R5421 prior to addition of ionomycin or DMSO (as control solvent for ionomycin, labeled "Control"). In each case, the data represent the mean ± SEM (n = 13–19). The change in light scatter intensity from the initial minimum to that observed 240 s later (*M*) was calculated as in Figure 2.

The role of phosphatidylserine exposure in calcium-stimulated microvesicle release was studied using the scramblase inhibitor, R5421. At the concentration used, R5421 reduced the amount of phosphatidylserine translocated to the outer leaflet of the cell membrane during calcium influx by more than 50% (see Figure 6A and Ref. [3]). Under such conditions, microvesicle release also appeared to be significantly inhibited by the drug (Figure 6B).

## Discussion

Our purpose was to characterize microvesicle release from ionophore-treated erythrocytes in terms of microscopic membrane physical properties such as lipid order, fluidity, and composition. Previous studies had shown that these properties could be manipulated in human erythrocytes by altering temperature [13], membrane cholesterol content [1,29], and by using the scramblase inhibitor R5421 [3]. Our observations support two conclusions. First, calcium-stimulated microvesicle shedding depends on the level of membrane fluidity. This conclusion is based on the strong two-dimensional correlation between microvesicle release and diphenylhexatriene fluorescence (Figure 5). The temperature dependence of microvesicle release observed here (Figure 2) also corresponds well with other reports of erythrocyte physical behavior in the range of 30 to 40°C [1,13,29,35-37]. The second conclusion is that the loss of membrane phospholipid asymmetry that accompanies calcium loading is necessary for microvesicle release.

As always, one must exercise caution in interpreting data with MBCD. Although the compound is used to manipulate membrane cholesterol content, it is also capable of extracting other lipids [38]. For this reason, we use relatively mild extraction conditions (30 min incubation, ≤ 1 mM MBCD) [1,2]. Nevertheless, we cannot exclude the possibility that some of the observations shown in Figures 4 and 5 involve lipids other than cholesterol. For this reason, our interpretations are based on relationships between the observed physical properties and membrane behavior rather than trying to draw direct conclusions about cholesterol *per se*. We were able to eliminate the possibility of non-specific direct artifacts of MBCD by including a control experiment in which we pre-loaded the MBCD molecules with cholesterol (see Ref. [2] for experimental details). Treatment of cells with pre-loaded MBCD had a significantly smaller effect on microvesicle shedding compared to treatment with virgin MBCD (not shown).

An alternative explanation for the temperature effect in Figure 2 is that it reflects direct thermal sensitivity of protein behavior independent of lipid properties. This possibility has been considered previously for the enzymes such as scramblase that mediate phosphatidylserine flip-flop [13]. In that case, no temperature sensitivity was identified that could account for the data of Figure 2. Moreover, direct effects of temperature on protein activity or conformation alone cannot explain the effects of MBCD shown in Figures 4 and 5. Finally, the data of Figure 3 exclude the possibility that the temperature dependence was due to variation in the ability of ionomycin to transport calcium.

How would the fluidity of the lipid bilayer affect the ability of the membrane to release vesicles? This question is further complicated by an apparent paradox. On the one hand, microvesicle release appears dependent on a membrane that is fluid and/or disordered at the time calcium is introduced to the cell interior (Figures 4 and 5). On the other hand, influx of calcium causes the membrane to become more ordered as the vesicles are shed [22]. Some evidence suggests that the quandary may be resolved by considering the spatial distribution of physical properties along the membrane surface. A study by Salzer *et al*. concluded that membrane liquid ordered domains (termed "lipid rafts" in the paper) are involved in the vesiculation process because the microvesicles are enriched in lipids and proteins typical of these domains [16]. Two-photon microscopy of laurdan-labeled erythrocytes suggests that the increased membrane order associated with calcium ionophore treatment is distributed unevenly across the membrane, perhaps representing an expansion of liquid-ordered domains [1,3,13,22]. Therefore, as temperature is increased or cholesterol is removed, the ordered domains propagated by ionophore treatment would be superimposed on a background of greater fluidity thus creating greater contrasts between these domains and the surrounding lipids (as observed in Ref. [13]). Perhaps, then, enhanced microvesicle shedding at high temperature or low cholesterol is explained by a more extreme diversity in lateral bilayer structure. Whether the enhanced membrane fluidity promotes microvesicle release directly or indirectly by altering the activity of membrane-bound enzymes cannot be distinguished by the experiments of this study.

The role of phosphatidylserine exposure in microvesicle release has been controversial. It is well known that the two events are coincident when erythrocytes are loaded with calcium [3,19,39]. Similar coincidence is also observed when erythrocytes are stimulated with lysophosphatidic acid [40]. Furthermore, blood cells from patients suffering from a bleeding disorder known as Scott syndrome neither translocate phosphatidylserine across the cell membrane nor release microvesicles in response to calcium ionophore [19].

Naturally, these observations of coincidence do not designate cause and effect. It is in the attempts to establish causal relationships that the controversy has emerged. On the pro side, microvesicle release in platelets appears to require translocation of phosphatidylserine [17]. A similar claim has been made for erythrocytes [15]. One study used dithioerythritol to suppress phospholipid scrambling and discovered that microvesicle release was likewise suppressed [20]. In contrast, in erythrocyte ghosts loaded with spermine, calcium induced microvesicle release successfully even though phosphatidylserine translocation was severely reduced [21]. Similarly, spermine-loaded ghosts from a Scott syndrome patient also released microvesicles in the complete absence of phosphatidylserine flip-flop [21].

Inhibition of phosphatidylserine translocation with spermine or dithioerythritol is unlikely to be specific. Hence, use of the specific scramblase inhibitor R5421 in the present study may simplify interpretations. It also allowed experiments to be done on intact erythrocytes rather than relying on ghosts, in which cytoskeletal and cytosolic components are altered or depleted. The data shown in Figure 6 seem to provide an unambiguous interpretation with respect to cause and effect. Therefore, phosphatidylserine translocation appeared to be required for microvesicle release.

## Conclusion

In summary, two conclusions are supported by the results of this study. First, calcium-stimulated microvesicle release depends on the level of membrane fluidity. Second, loss of trans-membrane phospholipid asymmetry is required for microvesicle shedding.

Physiologically, the results presented in this study may relate to the process of membrane blebbing during apoptosis. It has been suggested that the microvesiculation of erythrocyte membranes in response to calcium loading is a model for the blebbing that occurs as part of the apoptotic process [41]. If this concept is true, it is logical to ask whether apoptotic membrane blebbing requires increased membrane fluidity as described in this report for microvesicle release. The possibility has not yet been examined experimentally, but investigations demonstrating that apoptotic cell membranes are more fluid or disordered than those of healthy cells support the viability of the idea [42-47]. These bilayer changes occur early during apoptosis and could be the result of phosphatidylserine exposure on the external membrane face [42,48-52] or other apoptotic events [42]. Based on these observations and the data presented in this report, we propose the hypothesis that increased fluidity of the cell membrane during apoptosis may be a necessary step in preparation for cell membrane blebbing.
